# Weeping Wound, Disgruntled Gut and Fading Hunger: Acrodermatitis Enteropathica in an Infant

**DOI:** 10.7759/cureus.100465

**Published:** 2025-12-31

**Authors:** Sushantika Sushantika, Jyoti Sethi

**Affiliations:** 1 Dermatology, All India Institute of Medical Sciences, Rishikesh, Rishikesh, IND

**Keywords:** acrodermatitis enteropathica, diarrhea, inherited disorder, oral and perioral regions, psoriasiform reaction, skin erosions, zinc metabolism

## Abstract

Acrodermatitis enteropathica (AE) is a clinical disorder that manifests due to severe zinc deficiency, which can be either genetic or acquired. The acquired form has been reported in patients with poor dietary intake, alcoholism, chronic liver disease, malabsorption syndrome, sickle cell anemia, or chronic renal failure, which usually presents at a later stage in life and has similar clinical features to the inherited defect.

On the other hand, the genetic form is an autosomal recessive disorder, characterized by periorificial dermatitis, alopecia, and diarrhea caused by a defect in the SLC39A4 gene located on human chromosome 8, band 8q24.3, which impairs zinc absorption in the small intestine. Zinc is necessary for the functioning of many regulatory genes and enzymes; its deficiency presents with diverse manifestations in childhood, most commonly. Genetic testing is usually not available in many places, so the diagnosis is made clinically, along with measurement of zinc levels in serum or hair, and replenishing with supplements as soon as possible.

This case report also depicts a case of inherited acrodermatitis enteropathica in an infant, which was managed successfully with zinc supplementation.

## Introduction

Acrodermatitis enteropathica (AE) is a clinical disorder that can be both acquired and inherited, with similar clinical features of symmetrical, red, scaly, crusted, or vesicobullous lesions around the mouth (perioral), anus (perianal), eyes (periocular), and acral areas. It is also associated with diarrhea, poor appetite, and weight loss in the patient. The global incidence can range from around 1 to 9 per 1,000,000 people or 1 in 500,000 newborns, for congenital defects.

The genetic defect responsible for AE occurs due to mutation of the SLC39A4 gene located on human chromosome 8, specifically at band 8q24.3, which is responsible for the manufacturing of ZIP4 (zinc/iron-regulated transporter-like protein), a crucial zinc transporter responsible for importing zinc into cells, especially in the intestines, and maintaining cellular zinc levels [1]. Over 100 different mutations in the gene can lead to an AE AE-like presentation. Zinc is an essential co-enzyme in metal enzymes (like alkaline phosphatase); it is an important structural component of gene regulatory proteins, and it also has a function in the regulation of gene expression.

The disease usually presents itself during childhood, after weaning. Signs and symptoms in infancy can include diarrhea, mood changes, anorexia, and neurological disturbance. In toddlers and older children, zinc deficiency is characterized by growth retardation, alopecia, weight loss, and recurrent infections [2]. The diagnosis is made by history-taking and clinical examination of the patient and is treated with lifelong oral zinc supplementation, typically 1-3 mg/kg/day elemental zinc, using forms like zinc sulfate or gluconate, to correct severe zinc malabsorption [3].

## Case presentation

A two-month-old formula-fed infant born via normal vaginal delivery at term out of a consanguineous marriage presented to the dermatology department with complaints of widespread erosive lesions over the body for one month. The erosions were preceded by fluid-filled lesions over the feet, loss of appetite, and frequent passage of non-bloody, loose stool. Family history was significant for similar complaints in the maternal uncle, who died in infancy due to a lack of access to medical care, as per the parents.

The parents denied any history of drug intake, fever, or systemic symptoms. Her immunization status was up-to-date, and no developmental delay was noticed. Her mother could not breastfeed the child due to insufficient lactation since her birth.

**Figure 1:**
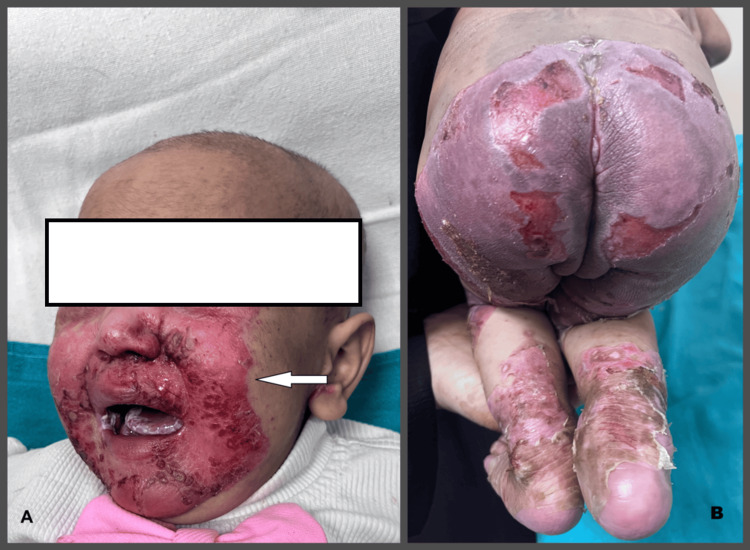
A) Facial lesions showing a well-demarcated erythematous plaque with periorificial scaling (marked with an arrow) and oral candidiasis; B) Erythematous plaque with scaling in the perianal area and lower legs

**Figure 2:**
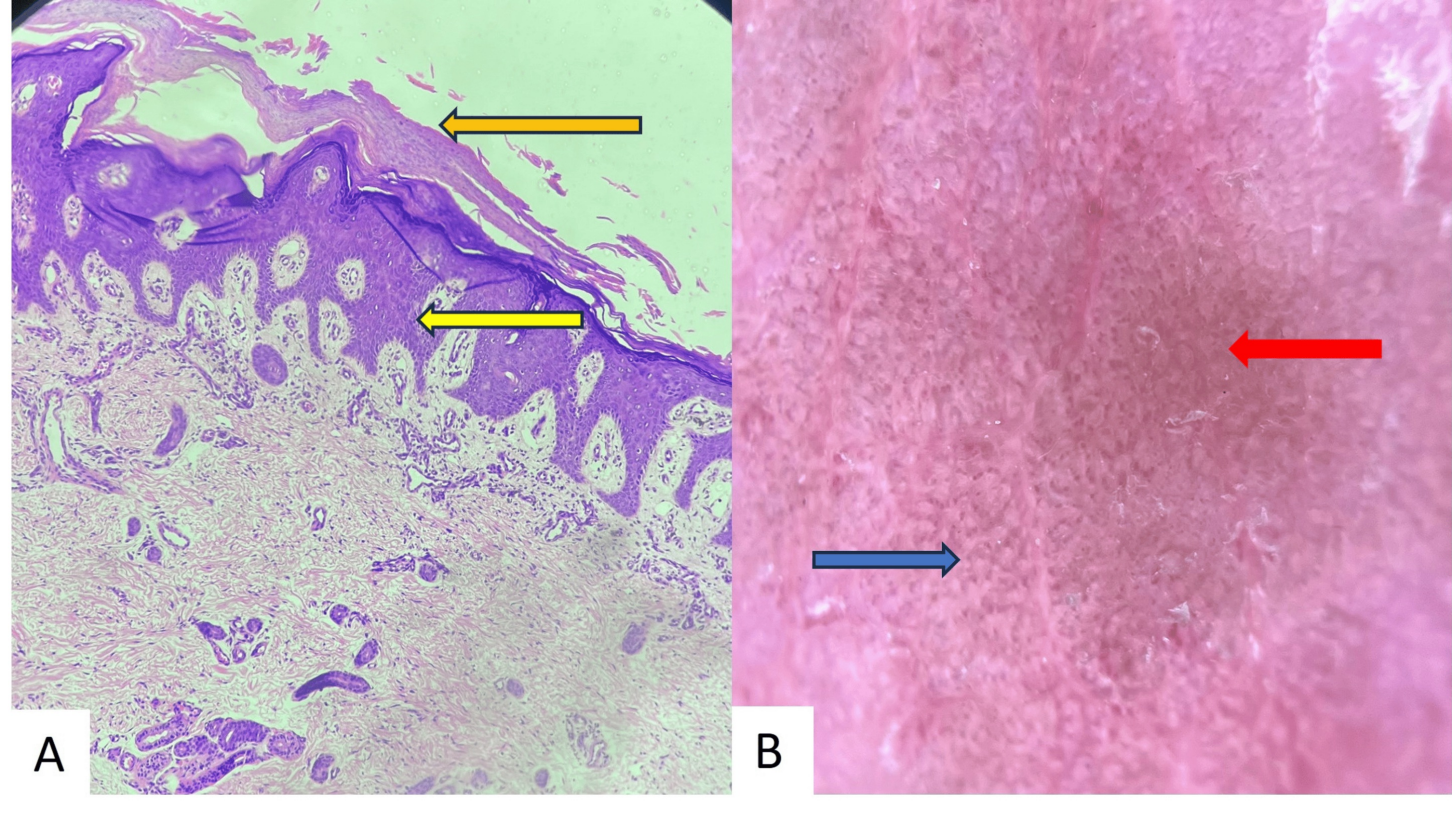
A) Regular acanthosis (yellow arrow) with confluent parakeratosis (orange arrow) (Hematoxylin & Eosin stain, 40x); B) Whitish scales, reddish-brown areas (red arrow), and red dotted vessels (blue arrow) (Dermlite DL200 Hybrid 10x)

On presentation, the child was irritable, with normal vital signs. Muco-cutaneous examination revealed multiple areas of denuded skin predominantly localized to periorificial areas, knees, hands, and feet, along with angular cheilitis (Figures 1A, 1B). The bedside Nikolsky sign was negative, and cutaneous cultures were sterile. Genetic testing could not be performed due to financial constraints. Dermoscopy of the lesion, along with a skin punch biopsy, was done from the gluteal site (Figures 2A, 2B).

Serum zinc levels were measured to be 20 µg/dL (normal value = 65-118 μg/dL) in the fasting sample, indicating a significant deficiency. Alkaline phosphatase was normal, along with other blood investigations. Histopathological examination showed psoriasiform reaction pattern with confluent parakeratosis, confirming our suspicion of acrodermatitis enteropathica.

**Figure 3:**
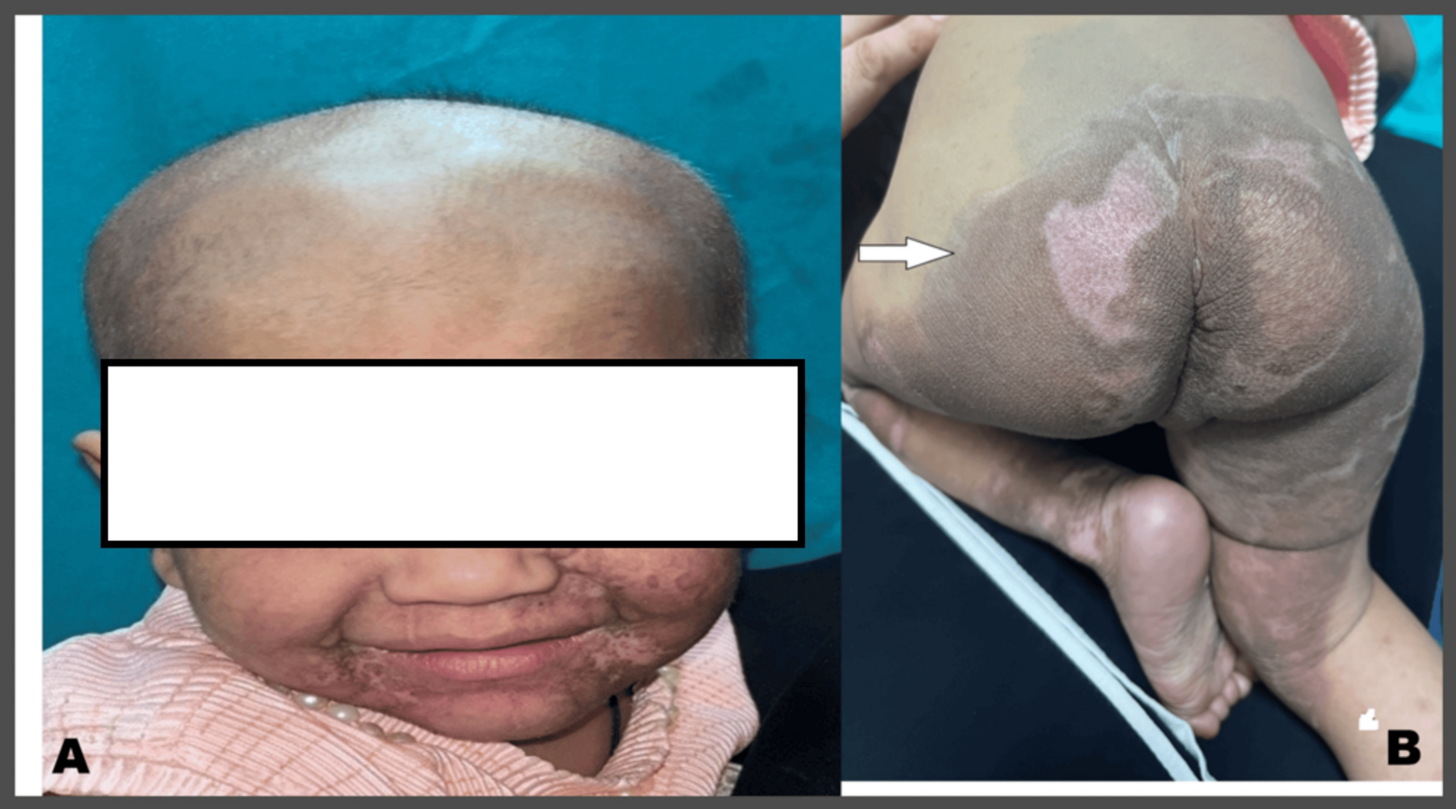
A) Healed lesions in the perioral area showing a healthy baby post-therapy; B) Similarly, lesions have healed in the perianal area as well (marked by the arrow)

With the clinical picture supported by histopathology and lab investigations, we considered congenital acrodermatitis enteropathica as the provisional diagnosis in this case, and the infant was started on oral zinc at a dose of 3 mg/kg/day. Ointment mupirocin with paraffin gauze dressing was advised for wound care. Clotrimazole mouth paint was advised for oral candidiasis. She showed significant improvement in two weeks with healing of denuded skin, clearance of mucosal candidiasis, and a decrease in the frequency of passage of stool (Figures 3A, 3B).

The patient was followed up monthly for three months; after full recovery, the dose of zinc was adjusted to 1 mg/kg body weight, and the mother was advised to follow up for routine checkups every three months and continue with oral zinc supplementation on a lifelong basis.

## Discussion

AE is a disorder of zinc deficiency that can be acquired or inherited. The inherited form is an autosomal recessive disorder caused by an SLC39A4 gene defect that affects zinc transport, which was first described by Danbolt and Closs in 1942 [4,5]. It is characterized by a triad of diarrhoea, acral and periorificial dermatitis, and alopecia. Cutaneous manifestations involve erythematous plaques, which can become vesicular, bullous, pustular, or desquamative. Other manifestations include neuropsychiatric features, apathy, loss of appetite, delayed puberty, growth retardation, developmental delay, and ocular complaints [6]. It is important to differentiate hereditary variants from acquired ones, which can be due to poor dietary intake, food faddism, intestinal abnormalities, premature birth, and a high catabolic state. Hereditary AE generally manifests after weaning or in formula-fed infants and persists lifelong with a positive family history in 30% of cases [7].

Depending upon sites of involvement, differential diagnosis of AE includes epidermolysis bullosa, *Staphylococcus* scalded skin syndrome (SSSS), pellagra, seborrheic dermatitis, widespread candidiasis, and toxic epidermal necrolysis (TEN). TEN is a severe mucocutaneous drug hypersensitivity characterized by targetoid lesions, which later lead to epidermal detachment. SSSS is a life-threatening infection caused by toxin-producing *Staphylococcus aureus*. It manifests as cutaneous exfoliation, widespread erythema, superficial desquamation, blistering, and a positive Nikolsky sign. Photodistribution of the exfoliative dermatitis in pellagra differentiates it from AE.

A literature search reveals there are many cases reported of congenital acrodermatitis enteropathica. In a report by Nicolai et al., the child diagnosed with AE had been admitted to hospitals multiple times due to recurrent infections and clinical symptoms diagnosed with lab evaluation and controlled with intravenous antibiotics and oral zinc supplementation [8].

Diagnosis of AE is generally clinical. Assessment of serum zinc levels, alkaline phosphate levels, and skin biopsy can help us reach a diagnosis. Histopathology is non-specific, presenting as a psoriasiform reaction pattern, epidermal pallor, and confluent parakeratosis [9].

Zinc is an essential element that plays a significant role in catalytic activity, structural integrity, regulatory function, and cell immunity [2]. Prolonged deficiency can result in growth delay, mental retardation, poor wound healing, and decreased immunity. Secondary infection caused by fungi and bacteria can lead to septicemia and death.

Treatment of AE requires 3 mg/kg/day of elemental zinc (50 mg of elemental zinc per 220 mg zinc sulfate) supplementation [3]. Patient’s plasma zinc levels should be monitored every three to six months with appropriate dose adjustment. Cultures should be obtained, and in cases with increased severity, an empirical antibiotic is recommended. Patients with AE show improvement within 24-48 hours on zinc supplementation [5].

## Conclusions

In conclusion, it is important to diagnose and treat such cases as soon as possible to prevent unnecessary morbidity. Differentiating AE from other diseases is also important to focus on the proper therapy within a reasonable period of time and provide immediate relief. Treatment is usually satisfactory in such cases, so early detection is the key to lifelong management.

Also, it is important to note whether the disorder is congenital or acquired, so that the parents can be advised for long-term supplementation of zinc accordingly.
